# Constructing and Validating Students’ Psychological Contract Violation Scale

**DOI:** 10.3389/fpsyg.2021.685468

**Published:** 2021-07-22

**Authors:** Yariv Itzkovich

**Affiliations:** Human Resource Management Department, School of Social Sciences and Humanities, Kinneret College on the Sea of Galilee, Zemach, Israel

**Keywords:** psychological contract, psychological contract violation, higher education, incivility, expectations

## Abstract

For two and a half decades, psychological contracts are researched mainly in work organisations as drivers of the attitudes and behaviours of employees, overlooking the importance of understanding the nature of the psychological contracts of students in higher education. This study constructs and validates a new scale for measuring the perceived psychological contract violations of students in the context of faculty incivility. A mixed-method approach was applied to study the issue in three phases. First, a qualitative method was used to capture and analyse the perceived entitlements of students, as described by 78 college students, resulting in 37 items or elements identified by students as reflecting their psychological contracts. Second, a sample of 244 students was studied to identify the perceptions of violated expectations of students. In the final phase, items were rephrased as expectations and were given to the third sample of 154 undergraduate college students to determine the level of fulfilment of these expectations. Additionally, to ascertain discriminate and convergent validity measures, students were asked about the extent to which they experienced faculty incivility (discriminant validity) and frustration with the quality of interaction with their faculty (convergent validity). From these results, students’ psychological contract violation scale was constructed and validated.

## Introduction

The term psychological contract is defined as the subjective perception of entitlements and obligations based on perceived promises (Robinson and Rousseau, 1994; Rousseau, 1995). Whether fulfilled or not, these perceived promises are rooted in social exchange theory, thereby driving both positive and adverse interrelations between individuals and organisations (Itzkovich and Heilbrunn, 2016). While psychological contract theory generally refers to work organisations and employee–employer relationships, in recent years, it has been applied by researchers investigating students’ expectations of higher education institutions, particularly the psychological contracts of specific sub-populations of students and their expectations concerning their advisors. Sub-populations of students that have been examined include master’s and Ph.D. students (Bordia et al., 2010); international students (Bordia et al., 2019); pharmacy students (Spies et al., 2010); student-athletes (Barnhill and Turner, 2015); and student volunteers (Haski-Leventhal et al., 2020). Some of these studies were qualitative (Koskina, 2013; Haski-Leventhal et al., 2020), and some used specific quantitative measures to capture the uniqueness of a target population, including the expectations of students learning online (Dziuban et al., 2015).

Although recent qualitative work has identified generic features of psychological contracts in higher education (Koskina, 2013), no research has applied a mixed-method approach to validate a generic quantitative measure of students’ psychological contract violation (SPCV). Understanding student expectations, namely, the components of the psychological contract, is crucial for planning, developing, and managing higher education systems, particularly as these institutions evolve into profit-driven systems, with students functioning as customers (Koskina, 2013).

Although Neoliberalism of higher education systems can be criticised for shifting towards the extraction of economic functions out of knowledge to adjust market demands (Del Cerro Santamaria, 2020), it is agreed that the marketisation of higher education train has left the station carrying marketisation logic in its carriages, to universities that adopt these trends similar to the private sector (Croucher and Lacy, 2020).

In this regard, students, the consumers in this equation, build on different expectations in a neoliberal context. Bunce and Bennett (2019) accounted for the theses neoliberal trends in higher education. The authors found that students who adopted consumer identity, similarly to paying customers, seize universities as service providers. As a result, these students feel entitled, choose courses that reflect maximisation of benefit, seek external utilities in their learning (compare to intrinsic motivation), and overall, adopt surface learning approaches that reflect their academic performance.

Failing to identify these expectations, namely students’ psychological contract components, could undermine higher education systems’ ability to meet those neoliberal expectations, thereby resulting in violations of these psychological contracts and, in market terms, losing the ability to compete.

Understanding these shifts does not necessarily mean abandoning the desire to promote deep learning. Gretzky and Lerner (2021) show us a way out. In their view, these expectations are divided into parallel and interrelated paths. The authors note that students do expect direct utilities but also emotional involvement in the process of learning. While the first set of expectations push higher education towards surface learning focussed on utilities, understanding the parallel set of expectations can help higher education adapt to these changes and maintain their essence. In this respect, higher education institutes can utilise the emotional needs translated to expectations to promote deep learning through students’ emotional engagement embedded in learning.

In turn, the cost of unfulfilled expectations is a critical element in research on psychological contracts, particularly their violations. While, as noted, the violation of psychological contracts has been studied in a broad organisational context, the research of psychological contract violation in higher education and its interrelations with other constructs has remained overlooked. No research has yet to examine the potential correlation between psychological contract violations and perceptions of faculty incivility (FI; Itzkovich et al., 2020), which can be expressed as lack of support, lack of fairness, and lack of positiveness, with support, fairness, and positiveness all vital components of student expectations (Koskina, 2013). Cultivating these components is crucial for the survival of higher education systems in the present age of supplier–customer relationships, which are increasingly shaping our higher education systems.

This current research can help the emerging, profit-driven higher education systems meet student expectations. Understanding student expectations have recently been shown to help reshape learning outcomes. Beenen and Arbaugh (2019) investigated student expectations from flipped classrooms and identified that students’ expectations, namely, psychological contract entitlements, significantly impacted flipped classrooms’ learning outcomes. Students who knew what to expect better coped with the flipped classroom methods, leading the study authors to suggest that institutions should clarify expectations prior to classroom engagement to manage the process better. In turn, prior explanations have been shown to reduce psychological contract violations (Rousseau, 1995). The meaning is that these explanations can help students adopt more realistic expectations, namely the components of students’ psychological contracts regarding their overall learning process. These explanations increase students’ emotional engagement in the learning process, which is also part of their expectations (Gretzky and Lerner, 2021).

Additionally, the current research will contribute to the study of incivility in higher education by clarifying the process of psychological contract violation underlying perceptions of FI (Itzkovich et al., 2020).

### Psychological Contract

The term psychological contract is defined as the subjective perception of entitlements and obligations based on perceived promises (Robinson and Rousseau, 1994; Rousseau, 1995). It refers to a set of entitlements and obligations that comprise an individual’s expectations in the workplace (Seter, 2001). Entitlements involve an individual’s expectations of positive outcomes either due to a particular social or organisational system or by merit of their contributions. The sense of entitlement is based on the individual’s expectations of achieving specific, defined results.

Conversely, obligations relate to the subjective duties that individuals feel towards a particular set of obtained resources attributed to their status in a social system or the rewards that they receive for that privilege (Seter, 2001). Until the 1990s, the psychological contract was considered the outcome of shared expectations resulting from a mutual dialogue between employees and organisations. Rousseau (1995) was the first to interpret the psychological contract as joint, but rather as the subjective perception of an individual concerning their expectations regarding giving and receiving in the workplace. The ground-breaking work by Rousseau (1995) emphasised that reciprocity or agreement between the parties to the psychological contract is not required for its creation. According to Rousseau (1995), such contracts are created, nourished, upheld, and even broken as reflections of the perception of the individual of the organisational reality. Rousseau (1995) also emphasised that the psychological contract is based on perceived pledges and is largely a conceptual construct whose elements are created by the understanding by an individual of what has been promised.

When psychological contracts are violated, people’s trust in the organisation is damaged, thereby diminishing their satisfaction with work and reducing their commitment to the organisation and their interest in remaining with the organisation over time (De Clercq et al., 2021). In faculty–student relationships, psychological contract violations can take many forms for each party to the contract. Faculty react differently when they feel that the psychological contract is violated by their unfulfilled expectations or reactions regarding student behaviour, such as disinterest or lack of respect. Some of these reaction patterns may foster an atmosphere of rudeness both within and beyond the classroom (Itzkovich et al., 2020).

Students’ reactions are similar. A student who feels that a faculty member has failed to fulfil that student’s core expectations, such as engaging thoughtfully or being attentive to student needs, might sense that the psychological contract has been violated. In turn, that student might react in a manner that the faculty member could perceive as a contract violation. Consequently, these adverse reciprocal reactions can be interpreted by students as FI.

### Faculty Incivility

Faculty incivility perpetrated is commonly directed against students or other faculty members (Clark, 2013; Goldberg et al., 2013; Itzkovich et al., 2020). While both types derive from the same source, the latter, faculty-to-faculty incivility, is considered a sub-category of workplace incivility. Both perpetrators and targets are employed within the same organisation. Indeed, faculty-to-faculty incivility and workplace incivility are expressed in similar behaviours, including giving colleagues or subordinates the silent treatment, micromanaging others, patronising others, and belittling the work of others (Wright and Hill, 2015).

Conversely, faculty-to-student incivility is more specific to academia and educational institutions and, together with student-to-faculty incivility, reflects potentially adverse interrelations between students and faculty.

Many definitions of incivility in academic settings describe it as an act of interference within a harmonious and cooperative learning atmosphere (Berger, 2000; Knepp, 2012). Academic incivility is also viewed in a broader sense as part of institutional incivility, or “repeated interpersonal mistreatment that violates institutional (including but not limited to academic institutes) and/or social norms of civil conduct” (Itzkovich et al., 2020, p. 20).

A survey on incivility in nursing education by Clark et al. (2009) examined uncivil behaviours within nursing schools. They found that faculty-to-student incivility ranged from generally disrespectful behaviours to poor classroom management and flexibility issues. Having identified the need for a more generic measurement, Alt and Itzkovich (2015) constructed and validated a tool to measure the more general phenomenon of FI. Their results corroborated the active (serious) as opposed to the passive (less severe or subtle) theoretical structure of academic incivility identified in previous research (Berger, 2000; Knepp, 2012).

### Psychological Contract Violation as a Process Underlying Perceived FI

The psychological contract mechanism is rooted in exchange relationships, whereby one party reciprocates the other’s contributions based on perceived gaps between expectations and fulfilment. These reciprocal interactions represent social exchange theory (SET) elements that posit that calculating cost and benefit is fundamental in human interactions (Itzkovich and Heilbrunn, 2016; Schilpzand et al., 2016). Extending beyond its economic exchange roots, the theory further proposes that individuals use social interactions to maximise their self-interests (either tangible, such as grades, or intangible, such as attention or respect). The emotional aspects inherent to all psychological contracts can be linked to the social exchange model of Blau (1964), which suggests that social exchange runs parallel to economic exchange in relationships between individuals. As the exchange of interpersonal relationships is part of all psychological contracts, an inappropriate exchange could violate the contract and, conversely, unfulfilled expectations could be interpreted as inadequate exchange, or specifically in academia, FI. If students feel that some of their core expectations, such as teachers being interesting, thoughtful, or attentive to student needs, are unfulfilled, they may feel that the psychological contract between them and faculty has been violated. Moreover, they might interpret a teacher’s uncivil behaviour as deliberate, perhaps even reciprocating in a manner that could be interpreted as student incivility towards faculty. Retaliatory actions between students and faculty promote incivility on both sides (Itzkovich et al., 2020).

### The Present Study

The goal of the present study was to construct and validate the efficient measurement of the violations of the student–faculty psychological contract, capturing the different dimensions of such violations. Data from undergraduate students in Israel were gathered in three phases by research assistants. A deductive-inductive approach was adopted for this scale development research, in which both logically derived categories and those that randomly arise from the data may find their way into the study (Merton, 1968; Strauss, 1987). This approach can determine the definition of a faculty-student contract violation while identifying additional meaningful categories pertinent to that definition.

## Materials and Methods

For all three study phases, participants were recruited by placing Internet ads in student forums inviting undergraduate students to participate in the research by completing a questionnaire. The purpose of the study was explained as examining student perceptions of the obligations of their teachers. Participant consent to complete the questionnaire was obtained, and the anonymity of participants was explicitly assured.

### Participants

Phase 1, a qualitative study designed to gather student expectations of operationalising the psychological contract in the educational setting, included 78 undergraduate students from one academic college (24% male and 76% female; 40% second-year students and 60% third-year students; 32 Jews, 36 Muslims, and 10 Christians).

In Phase 2, designed to identify the expectations violated, data were gathered from 244 undergraduate students with a mean age of 30.42 years (SD = 7.89), female (*n* = 102, 41.8%) and male (*n* = 112, 45.9.2%). Thirty participants did not report their gender. The year-of-study distribution was as follows: 14.8% first-year; 29.1% second-year; 45.1% third-year; and 11% fourth-year students. Regarding ethnicity, 41.6% were Jewish, 30.6% were Muslim, 14.3% were Christian, and 13.5% were Druze students.

Phase 3 participants represented a broad range of students, including 154 undergraduate students from five randomly selected academic institutions, with a mean age of 24.46 years (SD = 5.01). Participants self-identified as female (*n* = 84, 54.5%) and male (*n* = 54, 35.1%). The remaining 16 participants did not report their gender. The year-of-study distribution was as follows: 20.8% first-year; 35.5% second-year; 42.5% third-year; and 1.2% fourth-year students. Regarding ethnicity, 41.6% were Jewish, 30.6% Muslim, 14.3% Christian, and 13.5% Druze. The participants were enrolled in the following departments: Education (25.3%); Psychology (16%); Special Education (8.7); Economics (8%); Architecture (6.7%); Engineering (6.7%); Management (6%); Medicine (5.3%); Social Sciences (3.5%); Criminology (3.5%); Law (3.3%); Political Sciences (2%); Social Work (2%); and Physiotherapy (2%).

### Instrument and Procedures

Phase 1 employed an open-ended questionnaire to gather data. The participants were asked to describe, in their own words, the expectations (one or more) that they had of their lecturers. In Phase 2, the students were asked to review each item and indicate the extent to which they expected their lecturers to act as described (37 items). Additionally, they were asked to indicate whether their lecturers behaved as they had expected with respect to these 37 items. For example, (a) “I expect my teacher to give me high grades” was paired with (b) “My teacher gives me high grades.” Each item was given a Likert-type score ranging from 1 = *strongly disagree* to 5 = *strongly agree*.

In Phase 3 of the research, we asked participants to consider one of their courses and refer to it while answering the questions. The 30 items gathered in Phase 2 were phrased as perceived obligations, and participants were asked to answer the extent to which their lecturer fulfilled these obligations.

#### Perceived FI Scale

To test the discriminant validity of the scale, we used the perceived FI scale (PFIS) designed by Alt and Itzkovich (2015) and a distinct precursor similar to the psychological contract to measure the frequency of FI occurrences. The scale included two FI constructs: Factor I contained 13 items representing active FI (AFI), for example, “The teacher yells at you as a response to misunderstanding,” which was also considered an unfulfilled expectation for fair treatment. The second construct, Factor II, contained eight items pertaining to passive FI (PFI), for example, “The teacher ignores students’ questions during lectures.” Each item was given a Likert-type score ranging from 1 = *almost never* to 5 = *nearly always*. Internal consistency reliability is shown in Table 1.

**TABLE 1:** Result summary for measurement models.

Reflective variables	Convergent validity	Internal constituency reliability	Discriminant validity
	AVE	Cronbach’s Alpha	HTMT
	>0.50	> 0.70	Confidence interval does not contain 1
Faculty incivility	0.544	0.952	Yes
Psychological contract violation	0.594	0.962	Yes
Disappointment from relations with faculty (one item)	–	–	Yes

### Student Psychological Contract Violation

Based on Phase 2, respondents were asked to answer whether the lecturer fulfilled his or her obligations to the respondent. A sample question was, “To what extent did the lecturer in this course fulfil “his/her” obligation to treat you fairly?” Each item was given a Likert-type score ranging from the lecturer 1 = *has not fulfilled his/her obligation at all* to 5 = *has highly fulfilled his/her obligation*.

Additionally, one question, “To what extent did the quality of interaction with your lecturer disappoint you considering your initial expectations of the interaction?” was designed to test the general convergent validity of the model, in line with the guidelines of Hair et al. (2016).

Outer (measurement) model assessment was conducted according to the guidelines of Hair et al. (2016) prior to the assessment of the structural model, as reflected in Table 1.

## Results

### Phase 1—Qualitative Study

Two raters, experts in the research area of higher education learning environments and interrelations between individuals in academic and work contexts, analysed the answers given by students as short paragraphs independently. For interrater reliability, Kappa (k) (Cohen, 1960), commonly used in psychological research, was assessed. The k values were interpreted as follows: k < 0.20 poor agreement; 0.21 < k < 0.40 fair agreement; 0.41 < k < 0.60 moderate agreement; 0.61 < k < 0.80 good agreement; 0.81 < k < 1.00 very good agreement. Result of 0.61 < k < 1 was considered acceptable for the study’s current phase. Meeting the threshold also accounts for the quality of data that was generally clear and relevant. All descriptions lacking consensus (partially due to lack of clarity and irrelevancy) were discarded from the analysis.

Following this step, descriptions that were identified as too similar to other descriptions were also omitted. This part was performed through a discussion between the two raters. As a result of this process, the number of descriptions was reduced from 64 valid responses to 40. After this process was completed, the two raters coded the responses, compiled short items out of the coded text, and categorised the responses.

Specifically, following the inter-rater validation phase, the two raters coded the remaining texts by marking relevant words as appeared in the text without interpreting them as much as possible. The raters did not interpret the text to stay close as possible to the original text in line with the process suggested by Lindgren et al. (2020). The coded text was then compiled into short items.

Once compiled, the short items were divided into seven categories. As suggested by Lindgren et al. (2020). The qualitative data were divided into categories, not themes, as categories share a low degree of interpretation and are more descriptive. Thus, they fit better the short descriptions used in this research.

The compiled items were sent to two associate professors of Education and Psychology for screening. The experts were instructed to inspect the responses for their adherence to the suggested categories and their overall clarity. This resulted in the removal of three responses. At the end of this stage, 37 items collapsing into seven categories: (1) adapted teaching methods (eight responses); (2) fairness (four responses); (3) knowledge in student assessment (five responses); (4) supporting students (four responses); (5) in-depth knowledge of the course material (four responses); (6) personal characteristics (four responses); and (7) deviated expectations (eight responses), emerged.

The following example illustrates the process:

The original student statement:

This is the first year of my undergraduate degree. Sometimes I feel that the faculty speaks very fast. Due to my language difficulties (Hebrew is not my mother tongue), I don’t understand what is being said, and I would expect my teachers to be considerate and speak slower.

The codes marked: “language difficulties” and “expectations from teachers to be considerate.”

These codes were rephrased as “I expect my teacher to take into consideration my language difficulties.” The item was considered as part of the “supporting students” category.

### Phase 2—Capturing Violated Expectations

To capture the violated expectations, students were asked to review each item and indicate the extent to which they expected their lecturers to act as described, the extent their lecturers behaved as they had expected concerning each of these 37 items. Statistically, the sample mean score on the expectation was subtracted from the mean score on the actual behaviour of the lecturers as reported by the students. Outcomes that showed no difference between expectation and actual conduct and outcomes that showed that actual conduct exceeded expectations (i.e., no violation) were excluded from Phase 2 of the study. This analysis aimed to include only items in which actual behaviour did not meet expectations or those situations reflecting psychological contract violations. The whole process was aimed to identify items that students observe as meaningful (they are part of their expectations) and only those that potentially can be violated. Focussing on one of the seven omitted questions can illustrate the process. The omitted item was phrased as “I expect my teacher to express tolerance to my lateness.” This item was omitted either for being graded as not expected by students or for meeting or exceeding expectations. Concerning this item, the meaning is that faculty express tolerance to lateness as expected by students or more than expected (on average) and thus should not be included in the scale.

The procedure utilised in Phase 2 had excluded seven items: (1) I expect my teacher to express tolerance to my lateness, (2) I expect my teacher to be flexible concerning my absence, (3) I expect my teacher to smile during class, (4) I expect my teacher to have a sense of humour, (5) I expect my teacher to be nice, (6) I expect my teacher to be patient, and (7) I expect my teacher to listen to me during class. These items were not violated on average and could not differentiate between students who experienced violation and those whose expectations were met.

After excluding these items, we were left with a 30-item scale, hereinafter referred to as the student psychological contract violation for use in Phase 3.

### Phase 3—Validating the Final Scale

Exploratory factor analysis (EFA) was used to validate the seven factors found in Phase 1 of the study. A principal component analysis with varimax rotation was used to corroborate the stability of the SPCV structure (eigenvalue > 1.00; item loadings > 0.40). Following the EFA, the items were evaluated for evidence concerning content validity. At least three items had to be loaded0.40 or higher on every factor. To avoid collinearity, the loading of an item on a single factor was required to be more than0.15 apart from the loading of that item on another factor. The principal component analysis solution accounted for 77.38% of the variance and yielded only five categories, for which only four of the seven categories suggested by the content analysis were identified. Table 2 depicts the factor loadings of the EFA after item removal, with items in bold corresponding to the factor they load on. Using the criteria detailed above, seven items were removed based on content validity or for not having met the threshold. Table 2 shows the 23 items clarified after collapsing into the four subscales. All subscales were significantly correlated with each other.

**TABLE 2:** The SPCV EFA: factors, item descriptions, and item loadings (in bold).

Item description	Factors			
	F1	F2	F3	F4
	Fairness expectations	Teaching expectations	Deviant expectations	Knowledge expectations
1. The obligation to take into consideration your language difficulties	**0.804**			
2. The obligation to treat you fairly	**0.795**			
3. The obligation to allow you to ask questions during class	**0.780**			
4. The obligation to act morally towards you	**0.760**			
5. The obligation to allow you to participate during classes	**0.678**			
6. The obligation to write fair exams	**0.672**			
7. The obligation to build exams that fit your level	**0.636**			
8. The obligation to give you fair grades (grades that reflect your level)	**0.571**			
9. The obligation to focus your learning efforts prior to a test	**0.569**			
10. The obligation to use a variety of teaching methods		**0.789**		
11. The obligation to teach in a manner that encourages in-depth thinking		**0.720**		
12. The obligation to help you understand the material after class hours		**0.709**		
13. The obligation to support you to resolve learning difficulties		**0.679**		
14. The obligation to teach in an interesting manner		**0.648**		
15. The obligation to consider your needs		**0.634**		
16. The obligation to illustrate learning materials (i.e., by giving examples)		**0.576**		
17. The obligation to make sure you understood the material		**0.564**		
18. The obligation to raise your grades easily			**0.755**	
19. The obligation to give you high grades			**0.753**	
20. The obligation to help you during tests			**0.649**	
21. The obligation to demonstrate up-to-date knowledge in his/her courses				**0.898**
22. The obligation to be familiar with up-to-date literature in his/her courses				**0.832**
23. The obligation to demonstrate up-to-date knowledge regarding research related to the content he/she teaches				**0.815**

The final structure of the scale consisted of four factors: Factor I contained nine items representing *fairness obligations*; Factor II contained eight items dealing with the teacher’s *obligations to use adaptive teaching practices*; Factor III contained three items related to the teacher’s *obligations to be informed and knowledgeable*, and Factor IV contained three items dealing with *deviant obligations*.

**FIGURE 1:**
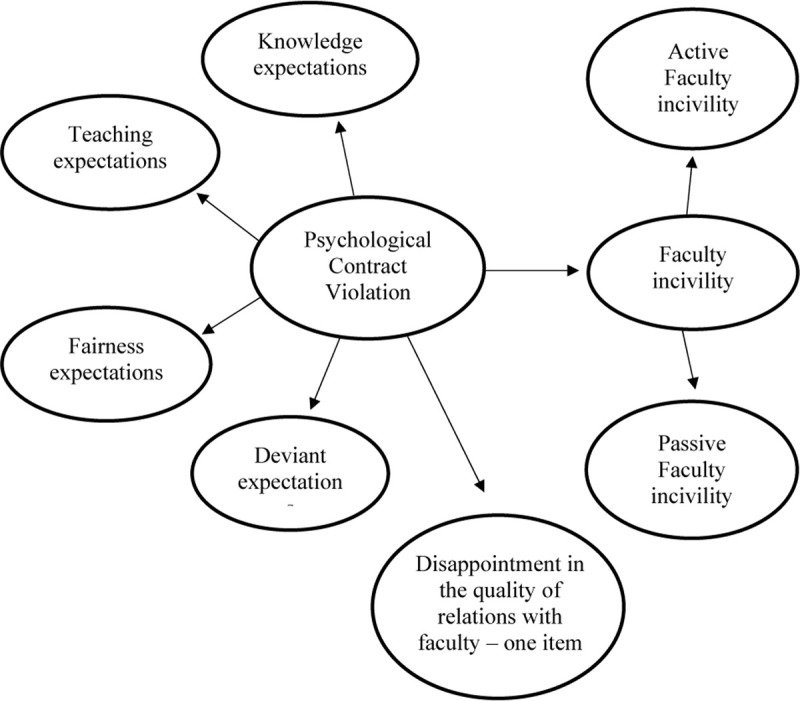
Research model.

To establish discriminant and convergent validity of the newly developed scale, we used the construct of FI as a discriminant validity criterion and the single general question formulated as a reflective measurement scale for psychological contract violation to test convergent validity. Figure 1 illustrates the model as tested using Partial Least Squares Structural Equation Modelling (PLS-SEM).

Discriminant validity was assessed using the heterotrait–monotrait ratio (HTMT) of the correlations (Henseler et al., 2015), defined as the mean of all correlations of indicators across constructs measuring different constructs. The HTMT serves as the basis for a discriminant validity test. An HTMT value above 0.90 suggests a lack of discriminant validity. Moreover, relying on a bootstrapping procedure, a bootstrap confidence interval containing a value of one indicates a lack of discriminant validity. The evaluation of Model 1 yielded satisfactory results. Namely, HTMT values ranged from 0.346 to 0.810, and the confidence interval did not include one, as shown in Table 1.

Moreover, in line with Hair et al. (2016), to assume convergent validity, the strength of the path coefficient between the newly developed scale and the single item scale had to exceed the threshold of 0.70. In the current model, as can be seen in Table 3, the path coefficient is equal to –0.799, thus proving that the convergent validity of the newly developed scale also measures psychological contract violation, as illustrated in Table 3. Overall, the four dimensions of SPCV explained 43.4% of the FI perceptions.

**TABLE 3:** Mean, STDEV, *T*-values, *P*-values.

	Original sample (O)	Sample mean (M)	Standard deviation (STDEV)	*T* statistics (|O/STDEV|)	*P* values
**Faculty incivility perceptions**					
Active Faculty Incivility	0.981	0.983	0.004	226.57	0.00
Passive Faculty Incivility	0.879	0.89	0.018	49.58	0.00
**Students’ psychological contract violation**					
Deviant Expectations	0.606	0.609	0.061	9.95	0.00
Disappointed	−0.799	−0.799	0.032	24.849	0.00
Faculty incivility perceptions	−0.661	−0.662	0.045	14.785	0.00
Fairness expectations	0.964	0.966	0.007	147.098	0.00
Knowledge expectations	0.689	0.694	0.052	13.198	0.00
Teaching expectations	0.951	0.954	0.008	113.989	0.00

## Discussion

This study aimed to construct and validate a generic scale for measuring the extent of the psychological contract violation of students, SPCV, and identifying its underlying components. The newly developed tool reflects the perceptions of students concerning their perceived entitlements concerning expected faculty obligations. The scale measures four dimensions of expectations: (1) fairness — the expectation that faculty will treat students fairly; (2) teaching — the expectation that faculty will use adaptive teaching practices and a variety of teaching methods; (3) knowledge — the expectation that faculty will be knowledgeable; and (4) deviant expectations — which refer to the assumption that faculty will help students get higher grades despite their lack of effort or prior knowledge.

The first dimension of student expectations relates to fair treatment from faculty. In this regard, students expect faculty to consider the challenges their students face, such as language barriers, that they will allow students to ask questions during class, and that, in general, the faculty will demonstrate high moral standards when teaching and evaluating student performance. These expectations were also found in the work of Koskina (2013), who noted that students held expectations of being taught by faculty who were “fair,” “honest,” “transparent,” and “supportive” (Koskina, 2013, p. 1,029). The underlying meaning of this finding is that the psychological contract of students is at least partially based on moral rather than professional expectations from the faculty.

The second dimension of the psychological contract relates to the quality of teaching. In this regard, students expect faculty to use various teaching methods, encourage in-depth thinking, and be interesting. This finding supports the finding by Koskina (2013) that faculty are expected to bolster their students’ abilities by, for example, building the self-esteem of students. Core competencies that are much in demand by organisations and employers, such as problem-solving skills, flexibility, and resilience, can be enhanced by using varied teaching methods (Itzkovich et al., 2020). In light of the rapidly changing workplace of today, there is a need for higher education institutions to provide their graduates with more tangible value that they can utilise in their future careers.

The third dimension of SPCV relates to faculty knowledge. This facet refers to the expectation for teachers to continually update their knowledge. Koskina (2013) also noted that faculty are expected to demonstrate excellent knowledge as part of the contract. The present study, however, phrased this expectation with a focus on the currency of learned material. Essentially, it implied that faculty members should continue to research their field of expertise to keep their knowledge up to date. To some extent, this requires faculty to balance between two different expectations: that they will invest in meaningful teaching and actively engage in research to stay up to date on their subject matter. The need for this balance was also noted by Itzkovich et al. (2020), who pointed out that the publish or perish culture might conflict with teaching tasks and thus distract from faculty investment in teaching.

The most surprising result of the current research was the finding that students also have deviant expectations, in that they expect faculty to raise student grades upon request, award high grades in general, and help students during tests. Altogether, these expectations shift the responsibility for the learning and achievements of the students from them to their faculty. Such expectations have not yet been examined in-depth, but they correspond to earlier findings concerning students’ expectations for high grades and their retaliatory responses to unmet expectations (Vaillancourt, 2013). It must be noted that such deviant expectations are contradictory to the goals of students of enhancing their competencies. Educational institutions are recommended to cultivate a culture in which faculty members can avoid meeting such deviant expectations while helping students understand the reasoning for this avoidance. Policies should teach students to take responsibility for their education to bolster their futures as lifelong learners.

A negative correlation was found between psychological contract fulfilment (i.e., the opposite of violation) and FI as part of the discriminant validity tests. This finding might suggest that perceptions of FI result from a process that starts when students begin to calculate the gap between their expectations and their fulfilment. Since some of their expectations are based on fairness, a gap between their expectations and their fulfilment is considered a violation of their psychological contract. That leads to an interpretation of faculty behaviour as incivility (Itzkovich et al., 2020). This finding means that an effort to avoid violation of the students’ psychological construct can mitigate the perception of FI, which remains an issue in higher education (Itzkovich et al., 2020).

This research has employed a mixed-method approach to validate the dimensions found in the qualitative work of Koskina (2013), resulting in a generic scaleable to capture student expectations and the extent to which they are perceived as having been fulfilled. In addition to the external validity, this study furthers research by investigating the expectations of different student populations. The findings are also consistent with previous work of Ampofo-Ansah et al. (2019), whose qualitative research found that a violation of the student psychological contract was attributable to a lack of support and poor faculty professionalism. These dimensions are thus embedded in the generic scale validated by the current research.

Recently Gretzky and Lerner (2021) investigated the neoliberal expectations of students in higher education. The authors found that the expectations of students are based on two complementary sets of expectations in the commercialised relationships between students and faculty that represent higher education institutes. The first set of expectations is based on instrumentality. Students expect pragmatism and self-fulfilment as a service they are entitled to receive as part of the economic exchange. They expect knowledge to be served in slices of pragmatic knowledge directly linked to their professional doing.

Providing emotional needs during the acquisition of knowledge is the second building block of the expected service. According to the authors, knowledge acquisition is expected to be provided in an emotionally engaging way. Beyond practice and practical tools, students expect to be emotionally involved in the process, feel it, be engaged with it, and consequentially, emotionally aroused. In the same but now negative vain, the authors demonstrate that unmet demand for correcting grades as an example is experienced as injustice.

Altogether the qualitative findings of the authors support the current quantitative results. The expectations for availability and support, the expectation for exciting and relevant knowledge accompanied by a variety of teaching methods, and the expectation for justice found in the current research is a reflection of the illuminating interpretation of Gretzky and Lerner (2021), who interpret the expectations of students as part of emotional capitalism in higher education—a mixture of straightforward pragmatism that is served in with an emotional touch.

## Practical Implications

Taking together, it seems that higher education institutes should strive to a new ethos of faculty in the neoliberal era. In a neoliberal higher education arena, faculty members must be more engaging, exciting, and updated. Additionally, faculty members should be more supportive and sensitive to answer student expectations. This is a significant change that requires a redesign of learning environments towards more constructivist pedagogy that is both engaging and supporting (Itzkovich et al., 2020).

As Gretzky and Lerner (2021) wrote, “despite the contradictions inherent to the incorporation of the business logic in the academic field and despite opposition to this move, utilitarian practices have been built into the relationship patterns in the university to the point of dominating them” (p. 13), and thus higher education institutes have no alternative but adaptation to answer their customers’ needs. At the same time, as long as institutions will manage to incorporate emotions into the academic processes, they will manage to navigate between students’ neoliberal expectations and higher education desire to promote knowledge *per se*. Specifically, incorporating an engaging emotional experience can balance the expectations for more instrumental learning and the desire of higher education to promote deep learning.

By doing so, higher education systems will also be able to balance between the deviant expectations of students as customers concerning their grades with the institutional need to lead and manage deep pedagogy without the need to negotiate grades that are perceived by customers as products. As long as the emotional need of students will be embedded in the pedagogical dialogue, these deviant expectations will be mitigated.

## Future Directions and Research Limitations

Although some of the questions in the new scale are based on a context where several different languages are spoken and thus can be considered a limitation, two separate forces support the generality of the scale.

Firstly, European Union (EU) expansion eastwards brought work immigrants to EU countries. These immigrants came from 12 different countries in Norway alone (Hoen, 2020). These immigrants who speak different languages shape multilanguage societies, not only in Norway.

Refugee migration is a complementary force that supports the generality of the scale. The vulnerability of refugees drives refugee migration. As forced immigration, it allows immigrants fewer opportunities with minimal choice (Brell et al., 2020). Thus, it is safe to assume that these two types of immigrants are accommodated in various countries.

All in all, these two complementary trends shape multilanguage societies in which language engages students and institutions in higher education. Although these trends support the generality of the scale, further research is needed to validate the scale and increase its external validity.

Although the need to expand the external validity of the scale developed throughout the current study contributes new insights into the complicated relationship between students and educational institutions as represented by faculty members. Understanding these delicate relations and clarifying student expectations is essential both for students’ achievements and for the success of educational organisations, especially in a neoliberal era when these expectations evolved dramatically.

## Data Availability Statement

The raw data supporting the conclusions of this article will be made available by the authors, without undue reservation.

## Ethics Statement

The studies involving human participants were reviewed and approved by Kinneret College Ethics Committee. The patients/participants provided their written informed consent to participate in this study.

## Author Contributions

The author confirms being the sole contributor of this work and has approved it for publication.

## Conflict of Interest

The author declares that the research was conducted in the absence of any commercial or financial relationships that could be construed as a potential conflict of interest.
